# Assessment of the Relation between the Expression of Oxaliplatin Transporters in Colorectal Cancer and Response to FOLFOX-4 Adjuvant Chemotherapy: A Case Control Study

**DOI:** 10.1371/journal.pone.0148739

**Published:** 2016-02-09

**Authors:** Bertrand Le Roy, Lucie Tixier, Bruno Pereira, Pierre Sauvanet, Emmanuel Buc, Caroline Pétorin, Pierre Déchelotte, Denis Pezet, David Balayssac

**Affiliations:** 1 CHU Clermont-Ferrand, Service de chirurgie et oncologie digestive, F-63003, Clermont-Ferrand, France; 2 CHU Clermont-Ferrand, Service d’anatomopathologie, F-63003, Clermont-Ferrand, France; 3 CHU Clermont-Ferrand, Délégation à la Recherche Clinique et à l’Innovation, F-63003, Clermont-Ferrand, France; 4 INSERM/UdA U1071, USC INRA 2018, M2iSH, F-63001, Clermont-Ferrand, France; 5 Université d’Auvergne, R2D2 – EA 7281, F-63001, Clermont-Ferrand, France; 6 INSERM U1107, Neuro-Dol, F-63001, Clermont-Ferrand, France; University of Kansas School of Medicine, UNITED STATES

## Abstract

**Background:**

Adjuvant chemotherapy for colorectal cancer is mainly based on the combination of 5-fluorouracil, folinic acid and oxaliplatin (FOLFOX-4). The pharmacological target of oxaliplatin remains intracellular and therefore dependent on its entry into cells. The intracellular distribution of oxaliplatin is mediated by organic cation transporters 1, 2 and 3 (OCT1, 2 and 3), copper transporter 1 (CTR1) and ATPase Cu^2+^ transporting beta polypeptide (ATP7B) and may modulate the efficacy of oxaliplatin-based chemotherapy. The aim of this study was to perform a retrospective study to assess the relation between the expression of oxaliplatin transporters in colorectal cancer before chemotherapy and the response to FOLFOX-4 adjuvant chemotherapy in responder and non-responder patients.

**Methods:**

This retrospective study was conducted at a single center (University Hospital of Clermont-Ferrand, France). The target population was patients with resectable colorectal cancer operated between 2006 and 2013. Inclusion criteria were defined for the responder patients as no cancer recurrence 3 years after the end of chemotherapy, and for the non-responder patients as cancer recurrence within 1 year. Other inclusion criteria were stages IIb–IV cancers, first-line adjuvant FOLFOX-4 chemotherapy, and the availability of resected primary tumor samples. Exclusion criteria were preoperative chemotherapy and/or radiotherapy, a targeted therapy, other anticancer drugs, cancer recurrence between the first and the third year after the end of chemotherapy and follow-up < 3 years. Immunostaining of oxaliplatin transporters (OCT1, 2, 3, CTR1 and ATP7B) and Ki-67 was assessed in tumor samples.

**Results:**

Retrospectively, 31 patients have been selected according to inclusion and exclusion criteria (15 responders and 16 non-responders). Before FOLFOX-4 regimen, OCT3 expression was significantly lower in responder patients compared to non-responders (p<0.001). According to multivariate analysis, OCT3 remains an independent criterion for adjuvant FOLFOX chemotherapy response (p = 0.039). No significant relation is reported between chemotherapy response and the expression of OCT1 (p = 0.49), OCT2 (p = 0.09), CTR1 (p = 0.45), ATP7B (p = 0.94) and Ki-67 (p = 0.34) in tumors.

**Conclusions:**

High expression of OCT3 could be an independent factor related to resistance to FOLFOX-4 chemotherapy.

## Background

The overall survival of patients with high-risk colorectal cancer (CRC) (stages II, III or IV) is improved by adjuvant chemotherapy [1,2]. First-line chemotherapy commonly associates 5-fluorouracil, folinic acid and oxaliplatin in the FOLFOX-4 protocol. However, nearly half the patients receiving adjuvant treatment do not benefit from oxaliplatin [2,3]. Platinum salts (e.g. oxaliplatin) form platinum adducts in DNA double strands, resulting in the inhibition of DNA replication. Consequently, the cellular distribution of platinum salts is considered to be a significant factor determining drug sensitivity [4]. Membrane transporters of xenobiotics are important factors involved in the intracellular distribution and efficacy of anticancer drugs [4]. *In vitro* studies have demonstrated that organic cation transporters OCT1, OCT2 and OCT3, belonging to the solute carrier family 22 (SLC22a1, SLC22a2 and SLC22a3, respectively), are involved in the cellular uptake of platinum salts, especially for oxaliplatin [5–7]. The Copper Transporter 1 (CTR1), belonging to the solute carrier family 31 (SLC31A1), is also linked to the influx of platinum salts such as oxaliplatin into cells, while its efflux can be linked to the copper transporter, ATPase Cu^2+^ transporting beta polypeptide (ATP7B) [8]. Thus, in the context of CRC, the tumor expression of OCT1, OCT2, OCT3, CTR1 and ATP7B may affect response to FOLFOX-4 chemotherapy. However, to this date, very little clinical studies have been done in CRC and no study in the context of adjuvant chemotherapy. In metastatic CRC, a high expression of OCT2 has been associated with a better prognostic and a high expression of ATP7B with a poor prognostic [9–11]. No clinical study has ever assessed the relation between the chemotherapy response and the expression of OCT1, OCT3 and CTR1 in CRC. The aim of this study was to perform a retrospective assessment, in tumor samples after surgery and before chemotherapy, of the relation between the expression of oxaliplatin transporters (OCT1, OCT2, OCT3, CTR1 and ATP7B) and the response to adjuvant FOLFOX-4 chemotherapy, in responder and non-responder patients.

## Methods

### Patients and study design

This retrospective study was conducted at a single center, the University Hospital of Clermont-Ferrand (France). The target population was patients with resectable CRC operated in the Digestive Surgery Unit of the University Hospital of Clermont-Ferrand (France) between 2006 and 2013. Inclusion criteria were defined for the responder patients as no cancer recurrence (discovered in imaging or colonoscopy) 3 years after the end of chemotherapy, and for the non-responder patients as cancer recurrence within 1 year after the end of chemotherapy. Other inclusion criteria were stages IIb, IIc, III or IV CRC, first-line adjuvant FOLFOX-4 (folinic acid, 5-fluorouracil and oxaliplatin) chemotherapy after CRC resection, and the availability of resected primary tumor samples. Exclusion criteria were preoperative chemotherapy and/or radiotherapy, a targeted therapy, other anticancer drugs, cancer recurrence between the first and the third year after the end of chemotherapy and follow-up < 3 years. Ethical approval was obtained from the Local Ethics Committee (CPP sud-est 6; IRB: 00008526). Considering the gravity of this type of disease (CRC), the absence of genetic exploration and in accordance with the French law, the Local Ethics Committee has given its approval for an exemption from the obligation to inform patients.

### Tissue samples and histopathology analysis

After tumor resection, fresh specimens were transported to the pathology laboratory, fixed in acetic acid/formalin, alcohol (AFA), embedded in paraffin, cut into 5-mm slices, and analyzed with HES stain (hematoxylin/eosin/safranin). Tumors were staged according to the tumor node metastasis (TNM) classification of the 7^th^ American Joint Committee on Cancer /Union for International Cancer Control. The following elements were also assessed for each tumor: degree of differentiation (poor, moderate or high), perineural invasion, vascular and/or lymphatic emboli, and margin status.

### Immunohistochemistry

Five-micrometer sections of paraffin-embedded tissue were labeled with rabbit polyclonal antibodies: anti-OCT1 (1:125, Novus biologicals, UK), anti-OCT2 (1:50, Atlas antibodies, USA), anti-CTR1 (1:200, Novus Biologicals, UK), anti-ATP7B (1:25, Abcam, France), monoclonal rabbit antibodies: anti-OCT3 (clone EPR6630, 1:300, Abcam, France) and anti-Ki-67 (clone MIB-1, ready-to-use, Dakko, Denmark). Ki-67 was used as a cellular marker for proliferation and a prognostic factor in CRC [12]. Immunostaining was performed with the Ultra View Detection Kit on a Benchmark XT stainer (Ventana Medical Systems, USA). The evaluation was performed independently by 2 experienced pathologists (authors: LT and PD) who evaluated OCTs, CTR1, ATP7B and Ki-67 expressions by light microscopy. OCTs and CTR1 were scored semi-quantitatively with an H-score, as described before [13]. Staining intensity was graded from 0 to 3+ (0: no staining; 1+: weak staining, 2+: moderate staining, 3+: strong staining). The percentage of stained tumor cells was evaluated for each intensity and an H-score (0–300) was calculated as follows: (1x percentage of cells harboring weak staining 1+) + (2x percentage of cells harboring moderate staining 2+) + (3x percentage of cells harboring strong staining 3+). ATP7B and Ki-67 were assessed by the percentage of stained cells. When there were discrepancies between the observers, the samples were reviewed until consensus was reached.

### Data management and statistical analysis

Study data were collected and managed using the REDCap (Research Electronic Data Capture) application hosted at the University Hospital of Clermont-Ferrand [14]. Statistical analysis was performed using Stata 13 software (StataCorp LP, College Station, USA). The tests were two-sided, with a type I error set at α = 0.05. It seemed difficult to propose a sample size estimation according to lack of knowledge on literature about the main objective and associated outcome in this population. Also, the sample size was fixed according recruitment ability and according to sequential design without type I error inflation [15]. However, *a posteriori* statistical power calculation was proposed. These aspects were clarified in discussion. Baseline characteristics were presented as mean (± standard-deviation) or median [interquartile range] for continuous data (assumption of normality assessed using the Shapiro–Wilk test) and as the number of patients and associated percentages for categorical parameters. Comparisons of patients’ characteristics between independent groups (chemotherapy’s response) were made using chi-squared or Fisher’s exact tests for categorical variables (gender, type of surgery, primary tumor localization, histological status, neurotoxicity grade), and the Student t-test or Mann-Whitney if assumptions of t-test were not met ((1) normality and (2) assumption of homoscedasticity studied using Fisher-Snedecor test) for quantitative parameters (age, body mass index (BMI), serum creatinine, tumor biomarkers, number of chemotherapy cycles, cumulative doses of oxaliplatin). The relationship between quantitative parameters was studied using correlation coefficients (Pearson or Spearman according to statistical distribution). A multivariate logistic regression model was used to study the relation between the expression of oxaliplatin transporters in chemotherapy response (dependent variable), by considering adjustments on relevant clinical parameters: tumor location, TNM classification, lymphatic and venous emboli, perineural invasion, number of isolated nodes, cumulative oxaliplatin doses and number of chemotherapy cycles. Due to the relative small sample size, a bootstrap estimation was considered to complete the multivariate analysis. The final model was validated by a two-step bootstrapping process. In each step, 1,000 bootstrap samples with replacements were created from the training set. In the first one, using the stepwise procedure, we determined the percentage of models including each of the initial variables. In the second step, we independently estimated the logistic regression parameters of the final model. The bootstrap estimations of each covariate coefficient and standard error of the mean were averaged from those replicates. An analysis using a ROC (receiver operating characteristic) curve was proposed and several indexes were then calculated (Youden’s index, Liu’s index, efficiency) to investigate the threshold value for optimizing the sensitivity and specificity of OCT3 expression to predict chemotherapy response. When appropriate (for the area under the ROC curve (AUC-ROC) and odds-ratio), 95% confidence interval were estimated. The AUC-ROC was compared to 0.75 [16].

## Results

### Patient characteristics

**Fig 1:**
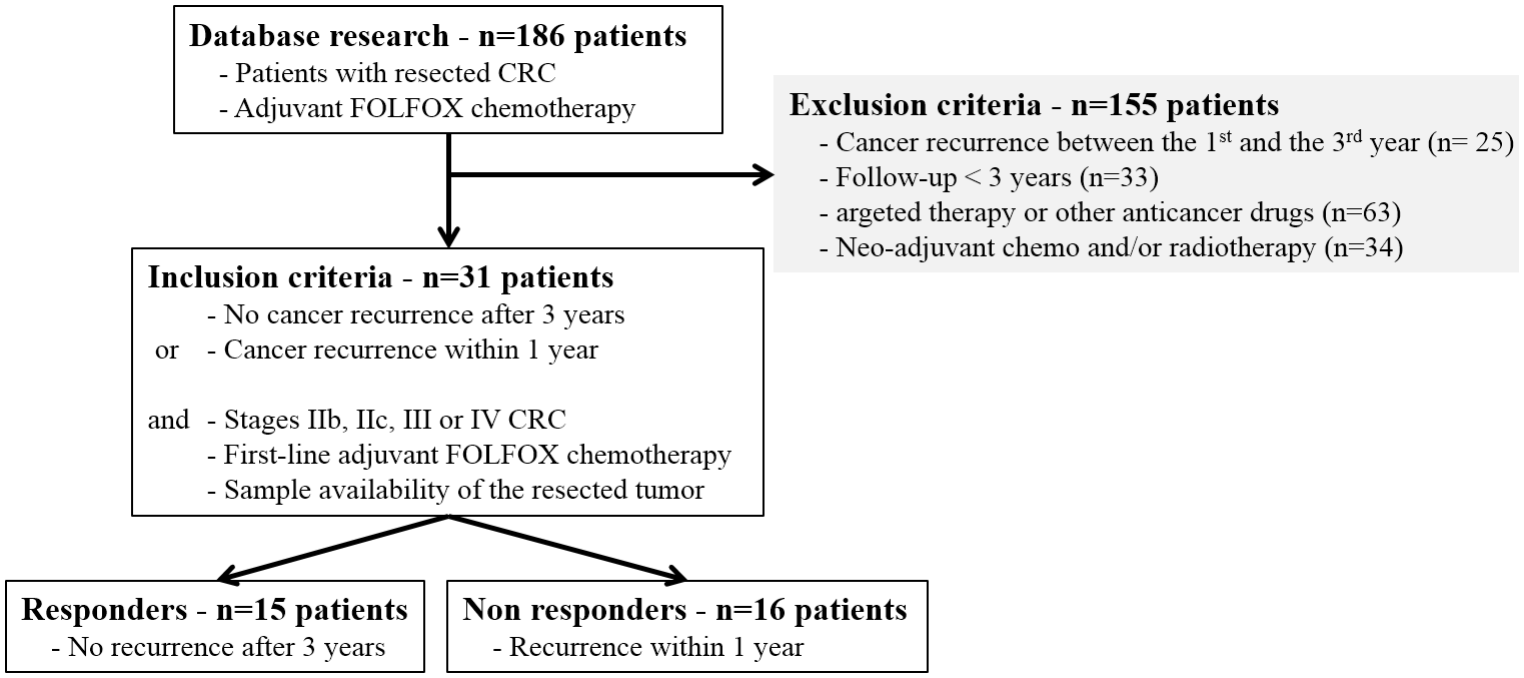
Flow Chart of patient selection. Patients were identified and selected from the database of the Digestive Surgery Unit between 2006 and 2013.

Retrospectively, 31 patients have been selected according to inclusion and exclusion criteria (Fig 1). Fifteen patients were identified as responders and 16 patients as non-responders. The clinical, pathological and therapeutic results of these 31 patients are summarized in Table 1. All patients were Caucasians and without any diagnosis of cancers in the last 5 years. No difference between the responder and non-responder patients was identified for age, gender, BMI, surgical procedure, post-operative complications, location of primary tumor, histological data, venous, lymphatic or nervous invasion, tumor differentiation, or tumor biomarkers. However, the number of chemotherapy cycles and the cumulative doses of oxaliplatin were significantly higher for the responder patients compared to the non-responders (p = 0.004 and p = 0.04, respectively).

**Table 1:** Clinical, pathological and therapeutic findings.

Items	Details	Responders	Non responders	p value
**Characteristics**	Number of patients	15	16	
	Men (men / women ratio)	11 (73%)	12 (75%)	0.92
	Age (years)	70.8 ± 10.4	69.9 ± 10.7	0.81
	BMI (kg/m^2^)	26.2 ± 3	27.9 ± 7.6	0.42
	Post-operative serum creatinine (μmol/L)	82.3 ± 15.6	71.1 ± 18	0.17
**Surgery**	Laparoscopy	1 (6.7%)	3 (18.8%)	0.58*
	Laparotomy	13 (87%)	11 (68.8%)	0.58*
	Laparoscopy conversion	1 (6.7%)	2 (12.4%)	0.58*
	Post-operative complications	3 (20%)	1 (6.3%)	0.33
**Primary tumor localization**	Right colon	5 (33.3%)	5 (31.3%)	0.12*
	Transverse colon	0 (0%)	0 (0%)	0.12*
	Left colon	5 (33.3%)	10 (62.5%)	0.12*
	Rectum	5 (33.3%)	1 (6.3%)	0.12*
**Histological status**	<pT3	4 (26.7%)	0 (0%)	0.07*
	pT3	10 (67.7%)	12 (75%)	0.07*
	pT4	1 (6.7%)	4 (25%)	0.07*
	pN0	1 (6.7%)	2 (12.5%)	0.36*
	pN1	11 (73.3%)	7 (43.7%)	0.36*
	pN2	3 (20%)	7 (43.7%)	0.36*
	pM0	14 (100%)	13 (81.3%)	0.23*
	pM1	0 (0%)	3 (18.7%)	0.23*
	Margin status R0	15 (100%)	16 (100%)	1
	Venous/ lymphatic emboli	5 (33.3%)	7 (43.7%)	0.55
	Perineural invasion	0 (0%)	4 (25%)	0.10
	Differentiation: poor	1 (7.1%)	1 (6.3%)	0.71*
	Differentiation: moderate	7 (50%)	11 (68.7%)	0.71*
	Differentiation: high	6 (42.9%)	4 (25%)	0.71*
**Tumor biomarkers**	CEA	4.08 ± 4.36	12.35 ± 25.05	0.56
	CA19.9	32.75 ± 47.08	27.46 ± 37.34	0.62
**Oxaliplatin-based chemotherapy**	Number of cycles	12 [12–12]	10 [6–12]	0.004
	Cumulative dose of oxaliplatin (mg/m^2^)	808 [711–878]	690 [430–782]	0.04
	Neurotoxicity	14 (93%)	11 (68.8%)	0.17
	Neurotoxicity Grade 1	10 (71.4%)	6 (54.5%)	0.43*
	Neurotoxicity Grade 2	3 (21.4%)	2 (18.2%)	0.43*
	Neurotoxicity Grade 3	1 (7.2%)	3 (27.3%)	0.43*

According to the findings, the results are expressed by number and ratio, mean ± standard deviation or median [interquartile]. The p-values are given for the comparison between responder and non-responder patients.

Metastasis status (pM) was unknown for 1 patient of the non-responder group and differentiation status was unknown for 1 patient of the responder group.

Metastatic patients (pM1) of the non-responder group had 1 liver metastasis.

Preoperative tumor biomarkers, carcinoembryonic antigen (CEA) and CA19.9, were available for 5 and 4 responder patients and for 8 and 5 non-responder patients, respectively.

* omnibus p-value for surgery: laparoscopy, laparotomy and laparoscopy conversion; primary tumor localization: right colon, transverse colon, left colon and rectum; histological status: <pT3, pT3 and pT4; pN0, pN1 and pN2; pM0 and pM1; tumor differentiation: poor, moderate and high; Neurotoxicity: grade 1, grade 2 and grade 3.

### Expression of OCTs, CTR1 and ATP7B transporters

**Fig 2:**
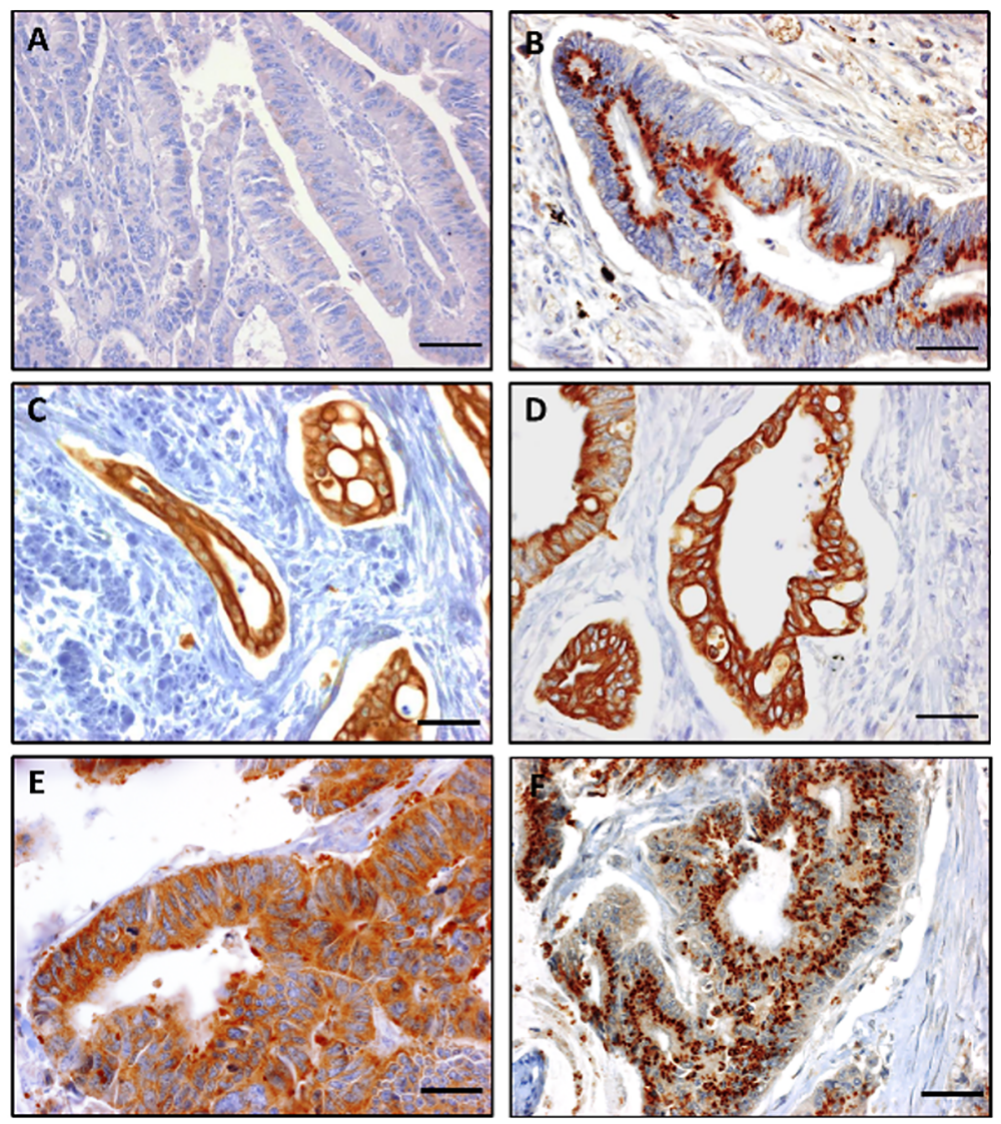
Immunostaining of oxaliplatin transporters on paraffin sections of colon tumors. Various intensity of immunostaining: (**A**) Negative control, (**B)** high expression (3+) of OCT1, (**C**) OCT2, (**D**) OCT3, (**E**) CTR1 and (**F**) ATP7B (x400; scale bar = 25μm).

**Fig 3:**
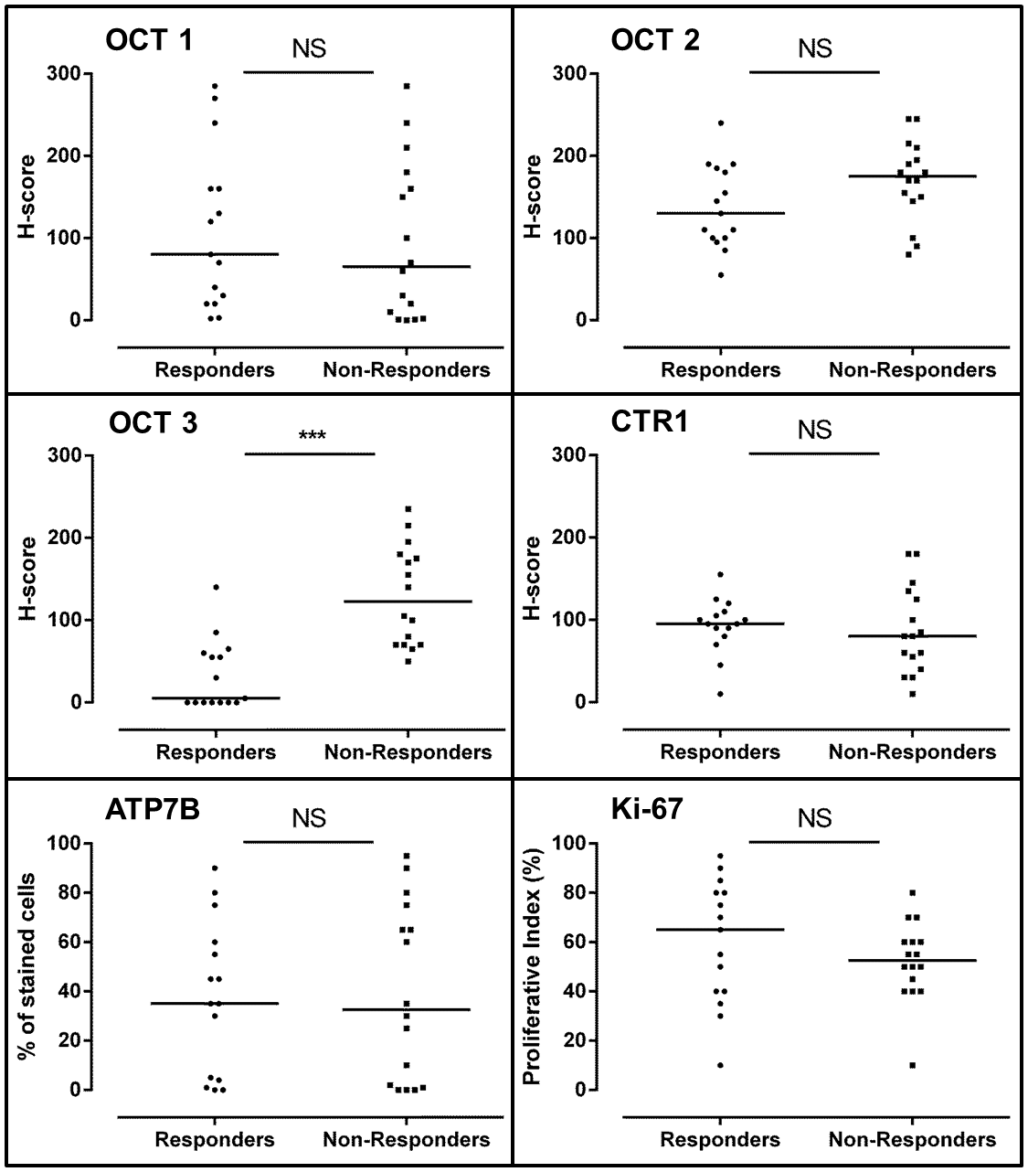
Distribution and median of H-scores for OCT1, 2, 3, CTR1, ATP7B and Ki-67 for responder and non-responder patients. (NS: not significant, p>0.05; ***: p<0.001).

**Fig 4:**
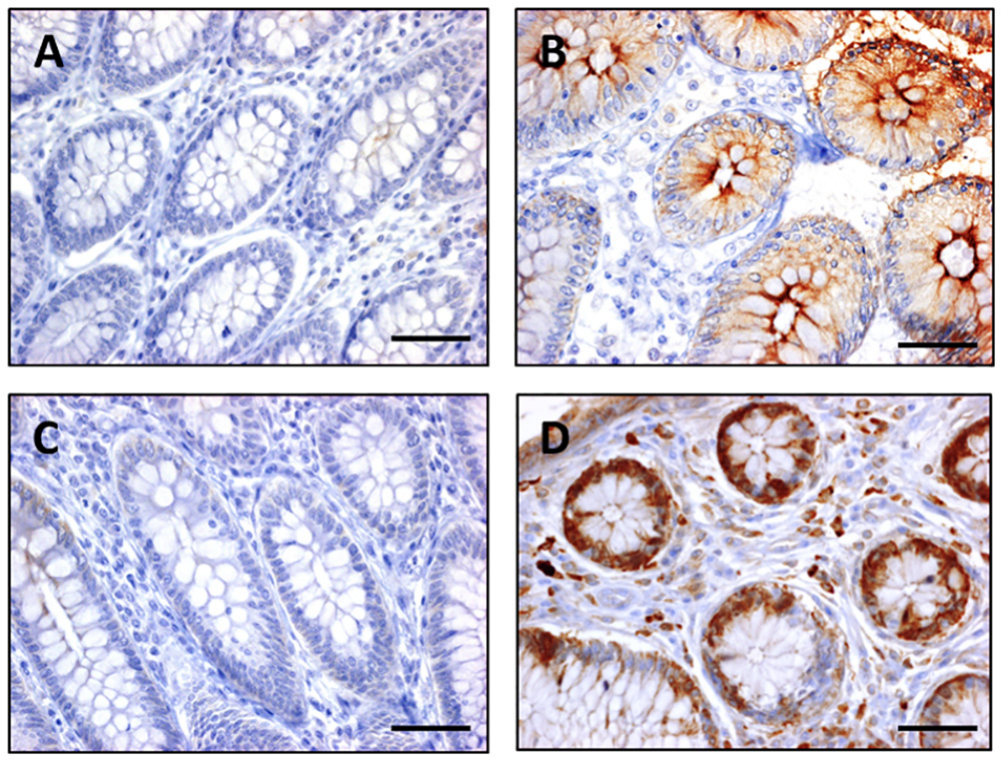
Immunostaining of OCT3 on paraffin sections of normal colon. Various intensity of immunostaining: (**A**) no expression in a responder patient, (**B)** high expression of a responder patient, (**C**) no expression in a non-responder patient, (**D**) high expression in a non-responder patient, (x400; scale bar = 25μm).

Staining of OCT1 and ATP7B revealed corpuscular subnuclear localization while OCT2 and OCT3 were located at the cytoplasmic membrane and also in the cytoplasm. CTR1 for its part is located in the cytoplasm (Fig 2). No significant difference of median H-score was found between responder and non-responder patients for OCT1 (80 [20–160] *vs* 65 [6–170], p = 0.49), OCT2 (130 [100–185] *vs* 175 [148–203], p = 0.09), CTR1 (95 [80–110] *vs* 80 [48–130], p = 0.45) and ATP7B (35 [4–60] *vs* 33 [2–70], p = 0.94) (Fig 3). For OCT3 expression in tumors, the median H-score was significantly lower for responder patients compared to non-responder patients (5 [0–60] *vs* 123 [70–178], p<0.001) (Fig 3). Expression of OCT3 in normal colonic tissue has been assessed in 5 samples of responder patients and 5 samples of non-responder patients. Compared to colonic tumor samples, the immunostaining intensity was low in normal tissue. No difference of OCT3 immunostaining has been put in evidence among group of patients (responder patients: 3 patients with no immunostaining and 2 patients with an immunostaining; non responder patients: 2 patients with no immunostaining and 3 patients with an immunostaining). Examples of OCT3 immunostaining in normal colonic tissue are shown in the Fig 4.

**Fig 5:**
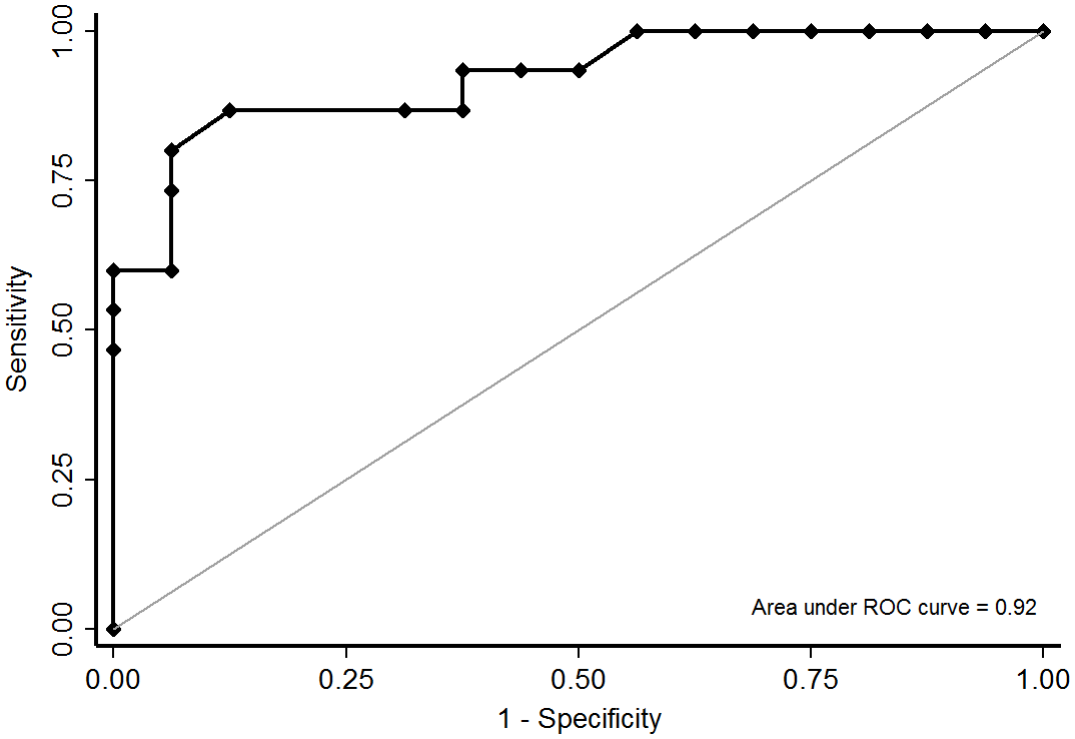
Receiver operating characteristic (ROC) curve of the association between OCT3 expression and FOLFOX-4 response. x-axis: 1-specificity, y-axis: sensitivity.

The relation between the expression of OCTs in tumors and response to FOLFOX-4 chemotherapy was assessed by multivariate analysis (considering the following variables: tumor location, TNM classification, lymphatic and venous emboli, perineural invasion, number of isolated nodes, cumulative oxaliplatin doses and number of chemotherapy cycles). Multivariate analysis showed that only low OCT3 expression in tumors preserved the independent prognostic significance of response to chemotherapy (odds ratio = 0.95 IC_95%_[0.91–0.98]; p = 0.039). After analysis with a ROC curve (AUC-ROC = 0.92 IC_95%_[0.82–1]) and determining the discriminant index for the H-score of OCT3, a threshold of 67.5 was found to be the most discriminating value between responder and non-responder patients (sensitivity = 87% IC_95%_[60%-98%], specificity = 88% IC_95%_[62%-98%]) (Fig 5). Considering this H-score threshold of 67.5, 86.7% (13/15) of responder patients were below this threshold against only 12.5% (2/16) of non-responder patients (p<0.001).

### Expression of Ki-67

No difference of Ki-67 expression has been found between responder patients and non-responders (65 [40–80] *vs* 52.5 [43.75–60], p = 0.34) (Fig 3). Ki-67 expression was not correlated to OCT3 expression in tumor samples (Spearman's rho = -0.12).

## Discussion

OCT1, OCT2, OCT3, CTR1 and ATP7B were detected by immunohistochemistry at various levels in CRC tissue samples after surgery and before adjuvant chemotherapy. Despite the small number of patients, high expression of OCT3 in tumor samples remained an independent factor of CRC non-response to adjuvant FOLFOX-4 chemotherapy. The expression of OCT1, OCT2, CTR1 and ATP7B were not related to FOLFOX-4 response. As it was mentioned in the statistical considerations (see above), due to difficulties to propose sample size estimation, *a posteriori* statistical power calculation was proposed according to results observed for primary outcome. With n = 15 responders and n = 16 non-responder patients, the statistical power was greater than 95% to show a difference of 118 points for OCT3 expression in tumors (H-score: 5 [0–60] *vs* 123 [70–178]), sampsi command of Stata software considering non-normal distribution [17]. This statistical power seemed satisfactory. For expression of OCT2, the statistical power was around 80% (H-score: 130 [100–185] *vs* 175 [148–203]). If the difference seemed clinically relevant for expression of OCT2, it was more ambiguous for expression of OCT1, CTR1 and ATP7B. As indicated by Feise [18], it must be careful to focus not only upon statistical significance, but also upon the quality of the research within the study and the magnitude of difference. Finally, the size-effect (difference between the means divided by standard deviation) for OCT3 expression between responders and non-responders was about 1.84 [0.98; 2.68], which can be considered as large (≥0.8) [19].

*In vitro*, the expression of OCT1, OCT2, OCT3 and CTR1 was positively related to the influx of oxaliplatin into cells and cytotoxicity while ATP7B was related to its efflux and resistance [5,7,8]. In the case of CRC, the relation between OCT1, OCT3 and CTR1 and chemotherapy response has never been assessed in clinical trials. Only two studies have observed a positive relation between OCT2 expression in metastatic CRC with better progression-free survival and tumor response [9,10]. However, comparison with our results is rather difficult, because only metastatic CRC patients were included, treated by FOLFOX-4 chemotherapy, with or without bevacizumab. The only clinical outcomes were progression-free survival and tumor response. Moreover, the number of patients treated with bevacizumab, chemotherapy cycles and cumulative doses of oxaliplatin was unknown [9,10]. The same comments can be made with the previous clinical study establishing a link between high ATP7B expression and a poor outcome in CRC patients receiving oxaliplatin-based chemotherapy [11]. This study included only metastatic CRC patients and treatment characteristics were not mentioned [11].

In view of the *in vitro* results of Yokoo *et al*., we could expect that the protein expression of OCT3 in CRC would be positively related to chemotherapy response [7]. OCT3 gene expression was significantly higher in CRC tissues than in normal tissues, and cells transfected with human OCT3 cDNA accumulated significantly more platinum than the empty-vector transfected cells [7]. However, *in vivo*, the exact function of OCT1, OCT2 and OCT3 in the cellular distribution of xenobiotics still remains unclear [20]. OCTs are facilitated diffusion bidirectional transporters of a wide range of xenobiotics and translocate their substrates down the electrochemical gradient across the membrane [20]. Based on literature, OCT2 seemed to be expressed on the basolateral side of enterocytes, OCT3 on the apical side and OCT1 on both sides [21–23]. In mice, OCT1 may facilitate the intestinal excretion of tetraethylammonium from blood to the intestinal lumen [24]. Also, recent works on metformin accumulation and secretion in salivary glands suggest that OCT3 can mediate both the influx and efflux of metformin in salivary glands [25]. OCT3 transporter in non-metastatic CRC could participate to the efflux of oxaliplatin from cancer cells, and finally, decrease the cytotoxicity of oxaliplatin in these cancer cells. But we must take precautions to interpret all these results, because extrapolation between *in vitro* and *in vivo* results is here contradictory. It would be interesting to assess in cultured cells and animal models of CRC expressing OCT3 transporter, the distribution and cytotoxicity of oxaliplatin, considering various expression or activity of OCT3 transporter. Even if these preliminary results must be confirmed, it could be interesting to discuss alternative strategy to overcome OCT3 overexpression in CRC patients. Irinotecan (CPT-11) has demonstrated a good efficacy in metastatic colorectal cancers [26,27] and would be an interesting candidate as a replacement of oxaliplatin, in patients with high expression of OCT3 protein. Irinotecan seems to be both a substrate and an inhibitor of OCT1 [28,29] and no information is actually available regarding its interaction with OCT3. In the same way, several drugs such as proton pump inhibitors can inhibit OCT3 transport [30], and consequently, would be able to increase oxaliplatin cytotoxicity in CRC.

Some criticisms can be made on our work. A quantitative assessment of gene and protein expressions on fresh tumor samples and correlation analyses would reinforce our results. In the same way, since 5-FU is also a key drug in the CRC chemotherapy, it would have been interesting to explore 5-FU and nucleoside transporters. For example, high expression of the human equilibrative nucleoside transporter 1 (hENT1) could be related to a poor clinical response in CRC [31]. In responder patients, number of FOLFOX-4 cycles and cumulative doses of oxaliplatin were higher than in non-responder patients (p = 0.004 and p = 0.04, respectively). These differences were explained by the treatment failure. For all the patients, responsiveness to chemotherapy was assessed after 6 cycles of FOLFOX-4 by computed tomography scans. For non-responder patients, the chemotherapy protocol has been changed from oxaliplatin to irinotecan (FOLFIRI) and for responder patients FOLFOX-4 chemotherapy was maintained until 12 cycles. Thereafter, in the non-responder patients, tumor sizes tended to be bigger (pT, responders *vs* non-responders, p = 0.07) and 3 patients of them were at a metastatic level. These 3 metastatic patients had only one liver metastasis and the primary and secondary tumors have been resected within the same surgical procedure. Pathological analysis of each resected lesions has reported a R0 resections. With a R0 resection of all metastatic disease, the 5-year overall survival has been reported to be as high as 58% [32]. The resection of metastatic liver remains the most efficacious strategy for achieving long-term overall survival and the only curative option for colorectal liver metastases especially with only one metastatic lesion [33]. Finally, the high OCT3 expression in this group could also be related to cancer aggressiveness or progression, but the multivariate analysis of OCT3 expression remained significant whatever the TNM status of patients, and no correlation has been put in evidence between OCT3 and Ki-67 expressions.

## Conclusion

This study is the first to address the relation between OCT3 expression in CRC and FOLFOX-4 response. In this retrospective clinical study, the expression of OCT3 in tumors was inversely related to chemotherapy response. Larger-scale and prospective study is needed to confirm these preliminary results and further *in/ex vivo* studies are required to elucidate the intimate role of OCT3 in the pharmacokinetics of platinum salts.

## Supporting Information

S1 Dataset (XLSX)

## Abbreviations

ATP7B: ATPase Cu^2+^ transporting beta polypeptide
BMI: body mass index
CRC: colorectal cancer
CTR1: Copper Transporter 1
FOLFOX: 5-fluorouracil, folinic acid and oxaliplatin
HES: hematoxylin/eosin/safranin
OCT1: Organic cation transporters 1
OCT2: Organic cation transporters 2
OCT3: Organic cation transporters 3
SLC: solute carrier family
TNM: tumor node metastasis
